# Diaqua­bis­(*N*,*N*′-diethyl­nicotinamide-κ*N* ^1^)bis­(4-fluoro­benzoato-κ*O*)copper(II)

**DOI:** 10.1107/S1600536811029941

**Published:** 2011-07-30

**Authors:** Hacali Necefoğlu, Füreya Elif Özbek, Vijdan Öztürk, Vedat Adıgüzel, Tuncer Hökelek

**Affiliations:** a Department of Chemistry, Kafkas University, 36100 Kars, Turkey; b Department of Physics, Hacettepe University, 06800 Beytepe, Ankara, Turkey

## Abstract

The asymmetric unit of the title mononuclear Cu^II^ complex, [Cu(C_7_H_4_FO_2_)_2_(C_10_H_14_N_2_O)_2_(H_2_O)_2_], contains one-half of the mol­ecule. The Cu^II^ ion is located on an inversion centre, and is coordinated by two N atoms from two diethyl­nicotinamide ligands, two O atoms from two 4-fluoro­benzoate (PFB) ligands and two water mol­ecules in a distorted octa­hedral geometry. In the PFB ligand, the carboxyl­ate group is twisted at an angle of 2.10 (14)° from the attached benzene ring. In the crystal structure, inter­molecular O—H⋯O hydrogen bonds link mol­ecules related by translation along the *a* axis into chains. Weak inter­molecular C—H⋯O hydrogen bonds and π–π inter­actions between the pyridine rings of neighbouring mol­ecules [centroid-to-centroid distance = 3.571 (2) Å] further consolidate the crystal packing.

## Related literature

For background to niacin, see: Krishnamachari (1974 ▶). For infomation on the nicotinic acid derivative *N*,*N*-diethyl­nicotinamide, see: Bigoli *et al.* (1972 ▶). For related structures, see: Hökelek *et al.* (1996 ▶, 2009*a* ▶,*b* ▶); Hökelek & Necefoğlu (1998 ▶, 2007 ▶); Necefoğlu *et al.* (2011 ▶). For bond-length data, see: Allen *et al.* (1987 ▶).
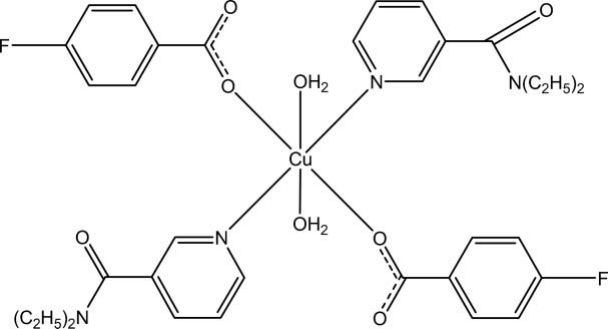


## Experimental

### 

#### Crystal data

[Cu(C_7_H_4_FO_2_)_2_(C_10_H_14_N_2_O)_2_(H_2_O)_2_]
*M* *_r_* = 734.25
Triclinic, 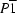

*a* = 7.4802 (2) Å
*b* = 8.6753 (2) Å
*c* = 14.6695 (4) Å
α = 77.164 (3)°
β = 84.723 (4)°
γ = 65.151 (2)°
*V* = 842.23 (4) Å^3^
*Z* = 1
Mo *K*α radiation
μ = 0.72 mm^−1^
*T* = 100 K
0.48 × 0.32 × 0.21 mm

#### Data collection

Bruker Kappa APEXII CCD area-detector diffractometer
Absorption correction: multi-scan (*SADABS*; Bruker, 2007 ▶) *T*_min_ = 0.759, *T*_max_ = 0.860
13620 measured reflections
4109 independent reflections
3714 reflections with *I* > 2σ(*I*)
*R*_int_ = 0.025

#### Refinement

*R*[*F* ^2^ > 2σ(*F* ^2^)] = 0.033
*wR*(*F* ^2^) = 0.099
*S* = 1.15
4109 reflections
233 parameters
2 restraints
H atoms treated by a mixture of independent and constrained refinement
Δρ_max_ = 0.57 e Å^−3^
Δρ_min_ = −0.45 e Å^−3^

### 

Data collection: *APEX2* (Bruker, 2007 ▶); cell refinement: *SAINT* (Bruker, 2007 ▶); data reduction: *SAINT*; program(s) used to solve structure: *SHELXS97* (Sheldrick, 2008 ▶); program(s) used to refine structure: *SHELXL97* (Sheldrick, 2008 ▶); molecular graphics: *ORTEP-3 for Windows* (Farrugia, 1997 ▶); software used to prepare material for publication: *WinGX* (Farrugia, 1999 ▶) and *PLATON* (Spek, 2009 ▶).

## Supplementary Material

Crystal structure: contains datablock(s) I, global. DOI: 10.1107/S1600536811029941/cv6646sup1.cif

Structure factors: contains datablock(s) I. DOI: 10.1107/S1600536811029941/cv6646Isup2.hkl

Additional supplementary materials:  crystallographic information; 3D view; checkCIF report

## Figures and Tables

**Table 1:** Hydrogen-bond geometry (Å, °)

*D*—H⋯*A*	*D*—H	H⋯*A*	*D*⋯*A*	*D*—H⋯*A*
O4—H41⋯O2^i^	0.83 (2)	1.90 (2)	2.7050 (19)	163 (3)
O4—H42⋯O3^ii^	0.83 (2)	2.01 (2)	2.834 (2)	172 (2)
C6—H6⋯O2^ii^	0.93	2.32	3.211 (2)	162
C10—H10⋯O2^iii^	0.93	2.48	3.394 (2)	170

Symmetry codes: (i) 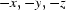; (ii) 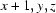; (iii) 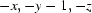.

**Fig. 1.**
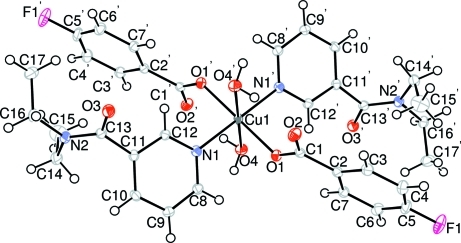
The molecular structure of the title molecule with the atom-numbering scheme. Displacement ellipsoids are drawn at the 50% probability level [symmetry code ('): -x, -y, -z].

## supplementary crystallographic information

### Comment

As a part of our ongoing investigations of transition metal complexes of nicotinamide (NA), one form of niacin (Krishnamachari, 1974), and/or the nicotinic acid derivative *N*,*N*-diethylnicotinamide (DENA), an important respiratory stimulant (Bigoli *et al.*, 1972), the title compound was synthesized. Herewith we report its crystal structure (Fig. 1).

The asymmetric unit of the title mononuclear Cu^II^ complex contains one-half molecule, the Cu^II^ atom being located on an inversion center. The unit cell of the title compound contains also two *N*,*N*-diethylnicotinamide (DENA) ligands, two 4-fluorobenzoato (PFB) ligands and two coordinated water molecules. All ligands coordinate the Cu in a monodentate manner. The crystal structures of similar omplexes of Cu^II^, Co^II^, Ni^II^, Mn^II^ and Zn^II^ ions, [Cu(C_7_H_5_O_2_)_2_(C_10_H_14_N_2_O)_2_] (Hökelek *et al.*, 1996), [Co(C_6_H_6_N_2_O)_2_(C_7_H_4_NO_4_)_2_(H_2_O)_2_] (Hökelek & Necefoğlu, 1998), [Co(C_9_H_9_O_2_)_2_(C_10_H_14_N_2_O)_2_(H_2_O)_2_] (Necefoğlu *et al.*, 2011), [Ni(C_7_H_4_ClO_2_)_2_(C_6_H_6_N_2_O)_2_(H_2_O)_2_] (Hökelek *et al.*, 2009*a*), [Mn(C_9_H_10_NO_2_)_2_(H_2_O)_4_].2H_2_O (Hökelek & Necefoğlu, 2007) and [Zn(C_7_H_4_BrO_2_)_2_(C_6_H_6_N_2_O)_2_(H_2_O)_2_] (Hökelek *et al.*, 2009*b*) have also been reported. In the copper(II) complex mentioned above the two benzoate ions coordinate to the Cu^II^ atom as bidentate ligands, while in the other structures all the ligands coordinate in a monodentate manner.

In the title complex,  four O atoms (O1, O1', O4 and O4', see Fig. 1) in the equatorial plane around the Cu^II^ ion form a slightly distorted square-planar arrangement, while the slightly distorted octahedral coordination is completed by the two N atoms of the DENA ligands (N1 and N1') in the axial positions. The near equalities of the C1—O1 [1.2716 (19) Å] and C1—O2 [1.247 (2) Å] bonds in the carboxylate groups indicate delocalized bonding arrangements, rather than localized single and double bonds. The Cu—O bond lengths are 1.9833 (11) Å (for benzoate oxygen) and 2.4192 (12) Å (for water oxygen), and the Cu—N bond length is 2.0192 (14) Å, close to standard values (Allen *et al.*, 1987). The Cu atom is displaced out of the mean plane of the carboxylate group (O1/C1/O2) by 0.7971 (1) Å. The dihedral angle between the planar carboxylate group and the adjacent benzene ring A (C2–C7) is 2.10 (14)°. The benzene A (C2–C7) and the pyridine B (N1/C8–C12) rings are oriented at a dihedral angle of A/B = 76.11 (6)°.

In the crystal structure, intermolecular O—H···O hydrogen bonds (Table 1) link the molecules related by translation along axis *a* into chains. Weak intermolecular C—H···O  hydrogen bonds (Table 1) and π–π interactions between the pyridine rings from the neighbouring molecules [Cg1···Cg1^i^ = 3.571 (2) Å; symmetry code: (i) 2 - x, 1 - y, -z; Cg1 is the centroid of N1/C8—C12] consolidate further the crystal packing.

### Experimental

The title compound was prepared by the reaction of CuSO_4_.5H_2_O (1.23 g, 5 mmol) in H_2_O (20 ml) and DENA (1.78 g, 10 mmol) in H_2_O (20 ml) with sodium 4-fluorobenzoate (1.62 g, 10 mmol) in H_2_O (50 ml) at room temperature. The mixture was filtered and set aside to crystallize at ambient temperature for a week, giving blue single crystals.

### Refinement

Atoms H41 and H42 (for water molecules) were located in a difference Fourier map and isotropically refined. The C-bound H atoms were positioned geometrically with C—H = 0.93–0.97 Å, and constrained to ride on their parent atoms, with *U*_iso_(H) = 1.2–1.5*U*_eq_(C).

### Crystal data

**Table:**

[Cu(C_7_H_4_FO_2_)_2_(C_10_H_14_N_2_O)_2_(H_2_O)_2_]	*Z* = 1
*M**_r_* = 734.25	*F*(000) = 383
Triclinic, *P*1	*D*_x_ = 1.448 Mg m^−^^3^
Hall symbol: -P 1	Mo *K*α radiation, λ = 0.71073 Å
*a* = 7.4802 (2) Å	Cell parameters from 9044 reflections
*b* = 8.6753 (2) Å	θ = 2.9–28.4°
*c* = 14.6695 (4) Å	µ = 0.72 mm^−^^1^
α = 77.164 (3)°	*T* = 100 K
β = 84.723 (4)°	Block, blue
γ = 65.151 (2)°	0.48 × 0.32 × 0.21 mm
*V* = 842.23 (4) Å^3^	

### Data collection

**Table:**

Bruker Kappa APEXII CCD area-detector diffractometer	4109 independent reflections
Radiation source: fine-focus sealed tube	3714 reflections with *I* > 2σ(*I*)
graphite	*R*_int_ = 0.025
φ and ω scans	θ_max_ = 28.5°, θ_min_ = 1.4°
Absorption correction: multi-scan (*SADABS*; Bruker, 2007)	*h* = −9→9
*T*_min_ = 0.759, *T*_max_ = 0.860	*k* = −11→11
13620 measured reflections	*l* = −19→19

### Refinement

**Table:**

Refinement on *F*^2^	Primary atom site location: structure-invariant direct methods
Least-squares matrix: full	Secondary atom site location: difference Fourier map
*R*[*F*^2^ > 2σ(*F*^2^)] = 0.033	Hydrogen site location: inferred from neighbouring sites
*wR*(*F*^2^) = 0.099	H atoms treated by a mixture of independent and constrained refinement
*S* = 1.15	*w* = 1/[σ^2^(*F*_o_^2^) + (0.0524*P*)^2^ + 0.2704*P*] where *P* = (*F*_o_^2^ + 2*F*_c_^2^)/3
4109 reflections	(Δ/σ)_max_ < 0.001
233 parameters	Δρ_max_ = 0.57 e Å^−^^3^
2 restraints	Δρ_min_ = −0.45 e Å^−^^3^

### Special details

**Table:**

Geometry. All esds (except the esd in the dihedral angle between two l.s. planes) are estimated using the full covariance matrix. The cell esds are taken into account individually in the estimation of esds in distances, angles and torsion angles; correlations between esds in cell parameters are only used when they are defined by crystal symmetry. An approximate (isotropic) treatment of cell esds is used for estimating esds involving l.s. planes.
Refinement. Refinement of F^2^ against ALL reflections. The weighted R-factor wR and goodness of fit S are based on F^2^, conventional R-factors R are based on F, with F set to zero for negative F^2^. The threshold expression of F^2^ > 2sigma(F^2^) is used only for calculating R-factors(gt) etc. and is not relevant to the choice of reflections for refinement. R-factors based on F^2^ are statistically about twice as large as those based on F, and R- factors based on ALL data will be even larger.

### Fractional atomic coordinates and isotropic or equivalent isotropic displacement parameters (Å^2^)

**Table:**

	*x*	*y*	*z*	*U*_iso_*/*U*_eq_	
Cu1	0.0000	0.0000	0.0000	0.01357 (9)	
O1	0.16849 (16)	−0.15368 (15)	0.10899 (8)	0.0158 (2)	
O2	−0.04849 (17)	−0.19328 (17)	0.21759 (8)	0.0203 (3)	
O3	−0.43885 (17)	−0.29279 (17)	−0.12427 (8)	0.0201 (3)	
O4	0.29700 (18)	0.01961 (18)	−0.07056 (9)	0.0209 (3)	
H41	0.242 (4)	0.068 (3)	−0.1223 (13)	0.047 (8)*	
H42	0.382 (3)	−0.074 (2)	−0.0817 (16)	0.032 (6)*	
N1	0.03309 (19)	−0.20678 (18)	−0.05247 (9)	0.0140 (3)	
N2	−0.2966 (2)	−0.34165 (19)	−0.26337 (10)	0.0170 (3)	
F1	0.65236 (17)	−0.32105 (18)	0.47171 (7)	0.0329 (3)	
C1	0.1133 (2)	−0.1884 (2)	0.19273 (11)	0.0144 (3)	
C2	0.2588 (2)	−0.2244 (2)	0.26753 (11)	0.0149 (3)	
C3	0.2080 (2)	−0.2568 (2)	0.36158 (11)	0.0177 (3)	
H3	0.0846	−0.2564	0.3777	0.021*	
C4	0.3405 (3)	−0.2895 (2)	0.43123 (12)	0.0209 (4)	
H4	0.3080	−0.3110	0.4941	0.025*	
C5	0.5214 (3)	−0.2892 (2)	0.40410 (12)	0.0212 (4)	
C6	0.5773 (2)	−0.2567 (2)	0.31234 (12)	0.0197 (3)	
H6	0.7008	−0.2568	0.2970	0.024*	
C7	0.4429 (2)	−0.2236 (2)	0.24351 (11)	0.0159 (3)	
H7	0.4761	−0.2006	0.1808	0.019*	
C8	0.2015 (2)	−0.3509 (2)	−0.04352 (11)	0.0158 (3)	
H8	0.3068	−0.3555	−0.0122	0.019*	
C9	0.2241 (2)	−0.4928 (2)	−0.07912 (11)	0.0170 (3)	
H9	0.3423	−0.5912	−0.0711	0.020*	
C10	0.0693 (2)	−0.4873 (2)	−0.12681 (11)	0.0159 (3)	
H10	0.0819	−0.5808	−0.1519	0.019*	
C11	−0.1058 (2)	−0.3376 (2)	−0.13609 (10)	0.0141 (3)	
C12	−0.1186 (2)	−0.2029 (2)	−0.09675 (11)	0.0141 (3)	
H12	−0.2374	−0.1052	−0.1011	0.017*	
C13	−0.2925 (2)	−0.3226 (2)	−0.17555 (11)	0.0148 (3)	
C14	−0.1364 (3)	−0.3544 (3)	−0.33111 (12)	0.0215 (4)	
H14A	−0.1352	−0.4266	−0.3732	0.026*	
H14B	−0.0117	−0.4107	−0.2979	0.026*	
C15	−0.1552 (3)	−0.1797 (3)	−0.38791 (16)	0.0354 (5)	
H15A	−0.0445	−0.1953	−0.4293	0.053*	
H15B	−0.1584	−0.1069	−0.3466	0.053*	
H15C	−0.2747	−0.1262	−0.4239	0.053*	
C16	−0.4830 (2)	−0.3304 (2)	−0.29629 (13)	0.0211 (4)	
H16A	−0.5377	−0.3920	−0.2465	0.025*	
H16B	−0.4550	−0.3885	−0.3486	0.025*	
C17	−0.6367 (3)	−0.1461 (3)	−0.32646 (15)	0.0288 (4)	
H17A	−0.7545	−0.1488	−0.3455	0.043*	
H17B	−0.5866	−0.0855	−0.3778	0.043*	
H17C	−0.6660	−0.0874	−0.2751	0.043*	

### Atomic displacement parameters (Å^2^)

**Table:**

	*U*^11^	*U*^22^	*U*^33^	*U*^12^	*U*^13^	*U*^23^
Cu1	0.01438 (14)	0.01390 (16)	0.01479 (14)	−0.00694 (11)	−0.00242 (9)	−0.00428 (10)
O1	0.0159 (5)	0.0163 (6)	0.0160 (5)	−0.0067 (5)	−0.0017 (4)	−0.0041 (4)
O2	0.0151 (6)	0.0250 (7)	0.0224 (6)	−0.0102 (5)	−0.0015 (4)	−0.0035 (5)
O3	0.0144 (5)	0.0262 (7)	0.0218 (6)	−0.0087 (5)	0.0012 (4)	−0.0092 (5)
O4	0.0163 (6)	0.0227 (7)	0.0216 (6)	−0.0052 (5)	0.0000 (5)	−0.0063 (5)
N1	0.0131 (6)	0.0153 (7)	0.0159 (6)	−0.0077 (5)	0.0011 (5)	−0.0044 (5)
N2	0.0141 (6)	0.0198 (7)	0.0189 (6)	−0.0069 (5)	−0.0020 (5)	−0.0063 (5)
F1	0.0312 (6)	0.0496 (8)	0.0231 (5)	−0.0225 (6)	−0.0122 (4)	−0.0007 (5)
C1	0.0147 (7)	0.0100 (7)	0.0197 (7)	−0.0047 (6)	−0.0021 (6)	−0.0052 (6)
C2	0.0156 (7)	0.0122 (8)	0.0176 (7)	−0.0056 (6)	−0.0011 (6)	−0.0045 (6)
C3	0.0167 (7)	0.0172 (8)	0.0206 (8)	−0.0080 (6)	0.0000 (6)	−0.0047 (6)
C4	0.0250 (9)	0.0235 (9)	0.0157 (7)	−0.0116 (7)	−0.0011 (6)	−0.0029 (7)
C5	0.0227 (8)	0.0231 (9)	0.0204 (8)	−0.0109 (7)	−0.0082 (6)	−0.0033 (7)
C6	0.0160 (8)	0.0209 (9)	0.0240 (8)	−0.0091 (7)	−0.0024 (6)	−0.0039 (7)
C7	0.0170 (7)	0.0148 (8)	0.0168 (7)	−0.0073 (6)	−0.0007 (6)	−0.0034 (6)
C8	0.0135 (7)	0.0177 (8)	0.0166 (7)	−0.0064 (6)	−0.0002 (5)	−0.0044 (6)
C9	0.0129 (7)	0.0174 (8)	0.0193 (7)	−0.0041 (6)	−0.0006 (6)	−0.0051 (6)
C10	0.0164 (7)	0.0144 (8)	0.0183 (7)	−0.0064 (6)	0.0011 (6)	−0.0065 (6)
C11	0.0141 (7)	0.0158 (8)	0.0149 (7)	−0.0082 (6)	−0.0002 (5)	−0.0037 (6)
C12	0.0126 (7)	0.0143 (8)	0.0162 (7)	−0.0059 (6)	−0.0008 (5)	−0.0036 (6)
C13	0.0145 (7)	0.0121 (8)	0.0188 (7)	−0.0055 (6)	−0.0020 (6)	−0.0037 (6)
C14	0.0196 (8)	0.0264 (10)	0.0183 (8)	−0.0075 (7)	0.0001 (6)	−0.0081 (7)
C15	0.0334 (11)	0.0348 (12)	0.0418 (11)	−0.0194 (9)	0.0083 (9)	−0.0077 (10)
C16	0.0186 (8)	0.0229 (9)	0.0255 (8)	−0.0082 (7)	−0.0068 (6)	−0.0099 (7)
C17	0.0203 (9)	0.0243 (10)	0.0397 (10)	−0.0036 (7)	−0.0097 (7)	−0.0097 (8)

### Geometric parameters (Å, °)

**Table:**

Cu1—O1	1.9833 (11)	C6—H6	0.9300
Cu1—O1^i^	1.9833 (11)	C7—C6	1.388 (2)
Cu1—O4	2.4192 (12)	C7—H7	0.9300
Cu1—O4^i^	2.4192 (12)	C8—H8	0.9300
Cu1—N1	2.0192 (14)	C9—C8	1.382 (2)
Cu1—N1^i^	2.0192 (14)	C9—C10	1.385 (2)
O1—C1	1.2716 (19)	C9—H9	0.9300
O2—C1	1.247 (2)	C10—H10	0.9300
O3—C13	1.2363 (19)	C11—C10	1.395 (2)
O4—H41	0.831 (17)	C11—C13	1.505 (2)
O4—H42	0.833 (16)	C12—C11	1.379 (2)
N1—C8	1.342 (2)	C12—H12	0.9300
N1—C12	1.343 (2)	C14—C15	1.514 (3)
N2—C13	1.339 (2)	C14—H14A	0.9700
N2—C14	1.467 (2)	C14—H14B	0.9700
N2—C16	1.475 (2)	C15—H15A	0.9600
F1—C5	1.3601 (19)	C15—H15B	0.9600
C1—C2	1.505 (2)	C15—H15C	0.9600
C2—C3	1.395 (2)	C16—C17	1.519 (3)
C2—C7	1.393 (2)	C16—H16A	0.9700
C3—C4	1.388 (2)	C16—H16B	0.9700
C3—H3	0.9300	C17—H17A	0.9600
C4—C5	1.376 (3)	C17—H17B	0.9600
C4—H4	0.9300	C17—H17C	0.9600
C6—C5	1.376 (2)		
			
O1—Cu1—O1^i^	180.00 (9)	C6—C7—C2	120.55 (15)
O1—Cu1—O4	85.97 (4)	C6—C7—H7	119.7
O1^i^—Cu1—O4	94.03 (4)	N1—C8—C9	122.39 (15)
O1—Cu1—O4^i^	94.03 (4)	N1—C8—H8	118.8
O1^i^—Cu1—O4^i^	85.97 (4)	C9—C8—H8	118.8
O4^i^—Cu1—O4	180.00 (9)	C8—C9—C10	119.52 (15)
N1—Cu1—O4	94.85 (5)	C8—C9—H9	120.2
N1^i^—Cu1—O4	85.15 (5)	C10—C9—H9	120.2
N1—Cu1—O4^i^	85.15 (5)	C9—C10—C11	118.13 (16)
N1^i^—Cu1—O4^i^	94.85 (5)	C9—C10—H10	120.9
O1—Cu1—N1	91.11 (5)	C11—C10—H10	120.9
O1^i^—Cu1—N1	88.89 (5)	C10—C11—C13	123.54 (15)
O1—Cu1—N1^i^	88.89 (5)	C12—C11—C10	118.98 (15)
O1^i^—Cu1—N1^i^	91.11 (5)	C12—C11—C13	116.96 (14)
N1^i^—Cu1—N1	180.00 (6)	N1—C12—C11	122.80 (15)
C1—O1—Cu1	127.30 (10)	N1—C12—H12	118.6
Cu1—O4—H41	91.8 (19)	C11—C12—H12	118.6
Cu1—O4—H42	114.0 (17)	O3—C13—N2	122.08 (15)
H41—O4—H42	102 (2)	O3—C13—C11	117.74 (14)
C8—N1—Cu1	122.34 (11)	N2—C13—C11	120.18 (14)
C8—N1—C12	118.14 (15)	N2—C14—C15	113.02 (15)
C12—N1—Cu1	119.51 (11)	N2—C14—H14A	109.0
C13—N2—C14	124.35 (14)	N2—C14—H14B	109.0
C13—N2—C16	117.12 (14)	C15—C14—H14A	109.0
C14—N2—C16	118.20 (14)	C15—C14—H14B	109.0
O1—C1—C2	115.78 (14)	H14A—C14—H14B	107.8
O2—C1—O1	126.09 (14)	C14—C15—H15A	109.5
O2—C1—C2	118.12 (14)	C14—C15—H15B	109.5
C3—C2—C1	119.85 (14)	C14—C15—H15C	109.5
C7—C2—C1	120.43 (14)	H15A—C15—H15B	109.5
C7—C2—C3	119.72 (15)	H15A—C15—H15C	109.5
C2—C3—H3	119.8	H15B—C15—H15C	109.5
C4—C3—C2	120.43 (16)	N2—C16—C17	114.09 (15)
C4—C3—H3	119.8	N2—C16—H16A	108.7
C3—C4—H4	121.1	N2—C16—H16B	108.7
C5—C4—C3	117.76 (15)	C17—C16—H16A	108.7
C5—C4—H4	121.1	C17—C16—H16B	108.7
F1—C5—C4	118.30 (15)	H16A—C16—H16B	107.6
F1—C5—C6	117.84 (16)	C16—C17—H17A	109.5
C6—C5—C4	123.86 (15)	C16—C17—H17B	109.5
C5—C6—C7	117.68 (16)	C16—C17—H17C	109.5
C5—C6—H6	121.2	H17A—C17—H17B	109.5
C7—C6—H6	121.2	H17A—C17—H17C	109.5
C2—C7—H7	119.7	H17B—C17—H17C	109.5
			
O4^i^—Cu1—O1—C1	−21.24 (14)	C14—N2—C16—C17	−93.55 (19)
O4—Cu1—O1—C1	158.76 (14)	O1—C1—C2—C3	177.38 (15)
N1^i^—Cu1—O1—C1	73.55 (14)	O1—C1—C2—C7	−1.8 (2)
N1—Cu1—O1—C1	−106.45 (14)	O2—C1—C2—C3	−1.9 (2)
O1—Cu1—N1—C8	−35.00 (12)	O2—C1—C2—C7	178.89 (15)
O1^i^—Cu1—N1—C8	145.00 (12)	C1—C2—C3—C4	−179.76 (16)
O1—Cu1—N1—C12	143.87 (12)	C7—C2—C3—C4	−0.6 (3)
O1^i^—Cu1—N1—C12	−36.13 (12)	C1—C2—C7—C6	179.96 (16)
O4^i^—Cu1—N1—C8	−128.95 (12)	C3—C2—C7—C6	0.8 (3)
O4—Cu1—N1—C8	51.05 (12)	C2—C3—C4—C5	−0.1 (3)
O4^i^—Cu1—N1—C12	49.92 (12)	C3—C4—C5—F1	179.97 (16)
O4—Cu1—N1—C12	−130.08 (12)	C3—C4—C5—C6	0.7 (3)
Cu1—O1—C1—O2	30.3 (2)	C7—C6—C5—F1	−179.76 (16)
Cu1—O1—C1—C2	−148.91 (11)	C7—C6—C5—C4	−0.5 (3)
Cu1—N1—C8—C9	179.77 (12)	C2—C7—C6—C5	−0.3 (3)
C12—N1—C8—C9	0.9 (2)	C10—C9—C8—N1	0.7 (2)
Cu1—N1—C12—C11	178.59 (11)	C8—C9—C10—C11	−0.7 (2)
C8—N1—C12—C11	−2.5 (2)	C12—C11—C10—C9	−0.8 (2)
C14—N2—C13—O3	171.65 (16)	C13—C11—C10—C9	−172.22 (15)
C14—N2—C13—C11	−8.5 (2)	C10—C11—C13—O3	118.97 (18)
C16—N2—C13—O3	−1.6 (2)	C10—C11—C13—N2	−60.9 (2)
C16—N2—C13—C11	178.22 (14)	C12—C11—C13—O3	−52.6 (2)
C13—N2—C14—C15	−89.5 (2)	C12—C11—C13—N2	127.53 (17)
C16—N2—C14—C15	83.7 (2)	N1—C12—C11—C10	2.5 (2)
C13—N2—C16—C17	80.1 (2)	N1—C12—C11—C13	174.46 (14)

Symmetry codes: (i) −*x*, −*y*, −*z*.

### Hydrogen-bond geometry (Å, °)

**Table:**

*D*—H···*A*	*D*—H	H···*A*	*D*···*A*	*D*—H···*A*
O4—H41···O2^ii^	0.83 (2)	1.90 (2)	2.7050 (19)	163 (3)
O4—H42···O3^iii^	0.83 (2)	2.01 (2)	2.834 (2)	172 (2)
C6—H6···O2^iii^	0.93	2.32	3.211 (2)	162
C10—H10···O2^iv^	0.93	2.48	3.394 (2)	170

Symmetry codes: (ii) −*x*, −*y*, −*z*; (iii) *x*+1, *y*, *z*; (iv) −*x*, −*y*−1, −*z*.
